# Effect of calcium salt of long-chain fatty acids and alfalfa supplementation on performance of Holstein bulls

**DOI:** 10.18632/oncotarget.23073

**Published:** 2017-12-09

**Authors:** Yang He, Wenjing Niu, Qinghua Qiu, Chuanqi Xia, Taoqi Shao, Haibo Wang, Qianwen Li, Zhantao Yu, Zhibiao Gao, Muhammad Aziz Ur Rahman, Huawei Su, Binghai Cao

**Affiliations:** ^1^ State Key Laboratory of Animal Nutrition, College of Animal Science and Technology, China Agricultural University, Beijing, China; ^2^ Gomal College of Veterinary Sciences, Gomal University, D.I. Khan, KPK, Pakistan

**Keywords:** calcium salt of long-chain fatty acids, alfalfa, rumen microbial flora, serum biochemical indexes, Holstein bulls

## Abstract

The purpose of this study was to assess the effects of calcium salt of long-chain fatty acids (CSFA) and alfalfa on beef cattle in the late fattening. 48 Holstein bulls were selected and randomly divided into 4 groups, feeding four dietary that *Leymus chinensis* with (LC) or with no (LN) 2.4% CSFA, and alfalfa replaced 50% *Leymus chinensis* with (AC) or with no (AN) 2.4% CSFA. The results indicated that alfalfa improved the feed conversion rate (*P* < 0.05). CSFA increased serum low density lipoprotein cholesterol, and reduced the cooking loss of Longissimus muscle (*P* < 0.05). CSFA and alfalfa reduced Acetate/Propionate. Alfalfa and CASF had significant additive effects on the apparent digestibility of dry matter, crude protein, neutral detergent fiber, acid detergent fiber, organic matter and rumen fermentation for acetate, isobutyrate, butyrate, isovalerate, total volatile fatty acids (*P* < 0.05). CSFA increased microbial diversity index when compared with alfalfa (*P* < 0.05), but no significant differences were detected in bacterial genera abundances among diets. The relative abundances of rumen bacterial genera have significant correlation with apparent digestibility of nutrients, rumen fermentation characteristics and serum biochemical parameters (*P* < 0.05). These results comprehensively evaluated the additive effects of alfalfa and CSFA on the application in Holstein bulls.

## INTRODUCTION

Holstein bulls had been often used to product meat as beef cattle in China. In order to improve the yield and quality of meat, ration often contains relatively high concentrates for beef cattle to deposit fat during the process of finishing. However, feed diets with high readily fermentable carbohydrates usually lead to subclinical acidosis of rumen and adverse to the long-time growth performance of beef cattle. Thus, by providing high-quality forage or adding oils to improve feed energy values could alleviate the rumen acidosis. On account of plant oils especially unsaturated fats are harmful to rumen micro-organisms [1], and bio-hydrogenation in rumen would transfer unsaturated fatty acids into saturated fatty acids [2]. Therefore, it is very necessary to use rumen-protected fat in the production.

CASF have been widely used in dairy as one of rumen-protected fats. It has been confirmed that the CASF can ameliorate energy negative balance for transition dairy heifers [3], increase the fat and dressing percentage of the beef [4]. Moreover, CASF can increase fiber digestibility and decrease rumen microbial bio-hydrogenation [5], thereby increasing the content of unsaturated fatty acids in milk and meat products, which are metabolically beneficial to human health. Ruminant liver metabolism is affected by the content and type of fatty acids in diet, ultimately change the composition of lipids in the blood [6].

As we know, the beef cattle growth performance and meat quality are influenced by diet factors. For example, alfalfa as a high quality roughage, provides protein in ruminant nutrition, can reduce the oxidation of fat and increasing the proportion of unsaturated fatty acids in the fat and improve meat quality of lambs [7, 8], rabbits [9] and pigs [10]. For example, diet supplement alfalfa can reduce cholesterol and increase polyunsaturated fatty acids in eggs [11], improve growth performance and carcass trait of lambs [8, 12]. Alfalfa flavonoids, extracted from alfalfa, can promote meat quality and antioxidant activity by regulating the lipid metabolism-related genes expression in adipose and liver tissues [13].

Although CASF and alfalfa have been studied on the ruminant nutrition respectively, previous research focused on their application in dairy cows. However, earlier studies did not from the rumen microbial to study growth performance and meat quality of Holstein bulls, especially the additive effects between CASF and alfalfa. Consequently, the aim of this study was to evaluate the effects of CASF, alfalfa and their additive effects on apparent digestibility, rumen microbial flora, serum biochemical indexes, and meat quality of Holstein bulls.

## RESULTS

### Alfalfa enhances growth performance and additive effect with CSFA on apparent digestibility of nutrients

The effects of diets on growth, feed intake and nutrients apparent digestibility are shown in Table 1. Replacement *Leymus chinensis* with alfalfa significantly increased feed conversion ratio and average daily gain (*P* < 0.05). The apparent digestibility of dry matter (DM), acid detergent fiber (ADF) and organic matter (OM) in AN group was significantly higher than that in LN and AC group. And the apparent digestibility of DM and OM in LC group was significantly higher than that in LN group (*P* < 0.05). The apparent digestibility of crude protein (CP) and neutral detergent fiber (NDF) in LC and AN diet were significantly higher than that in LN and AC diet (*P* < 0.05). There were significant additive effects between alfalfa and CSFA on apparent digestibility of DM, CP, NDF, ADF and OM (*P* < 0.05).

**Table 1:** Effect of dietary treatments on growth performance and digestibility of nutrients in Holstein bulls

Item		Dietary treatment				SEM	*p*-value			
		LN	LC	AN	AC		Treatment	alfalfa	CSFA	alfalfa × CSFA
Dry matter intake kg/d		12.37	12.96	12.59	12.48	0.132	0.411	0.623	0.367	0.178
Feed conversion ratio		11.23a	11.25a	8.87b	9.49b	0.203	<0.001	<0.001	0.380	0.410
Average daily gain		1.21a	1.16a	1.42b	1.39b	0.083	0.001	<0.001	0.412	0.897
Apparent digestibility of nutrients, %										
DM	67.1a		72.09bc	72.67c	69.17ab	0.605	0.002	0.255	0.518	0.001
CP	64.91a		72.44b	72.28b	66.68a	0.665	<0.001	0.508	0.424	<0.001
NDF	56.17a		64.95b	66.75b	56.06a	1.01	<0.001	0.648	0.604	<0.001
ADF	47.53a		53.65ab	57.43b	49.57a	1.264	0.026	0.240	0.725	0.005
EE	62.95		69.13	69.56	64.93	1.459	0.301	0.680	0.789	0.066
OM	75.51a		80.39bc	81.62c	77.62ab	0.651	0.003	0.182	0.725	0.001

LN = Leymus as the only roughage with No calcium salts of fatty acid, LC = Leymus as the only roughage with Calcium salts of fatty acids, AN = Alfalfa replace 50% leymus with No calcium salts of fatty acids, AC = Alfalfa replace 50% leymus with Calcium salts of fatty acids.

OM, organic matter; CP, crude protein; NDF, neutral detergent fiber; ADF, acid detergent fiber; EE, ether extracts; DM: dry matter.

*p*-value: Treatment: *P* value of Duncan’s multiple comparisons between diets treatments and different lowercase letters within the same row denote significant differences between the treatments; alfalfa, CASF and alfalfa × CSFA: the factorial analysis of crude protein levels, CSFA and their interaction.

#### CSFA increases serum low-density lipoprotein cholesterol concentrations

As shown in Table 2, added CSFA in feed significantly increased concentrations of serum low-density lipoprotein cholesterol (LDL-C) (*P* < 0.05), and tended to increase cholesterol (CHO) levels (*P* = 0.062). Serum alkaline phosphatase in AC group was significantly higher than that in LC group (*P* < 0.05). Replacing *Leymus chinensis* with alfalfa had a tendency to increase the activity of alkaline phosphatase (ALP) (*P* = 0.080). Serum urea concentration in LC group was significantly higher than that in LN and AC group (*P* < 0.05). There was significant additive effect between alfalfa and CSFA on serum urea (*P* < 0.05).

**Table 2:** Effects of dietarys on serum biochemical parameters of Holstein bulls

Item	Dietary treatment				SEM	*p*-value			
	LN	LC	AN	AC		Treatment	alfalfa	CSFA	alfalfa×CSFA
Glucose (mmol/L)	5.14	4.94	4.99	4.89	0.097	0.831	0.635	0.450	0.809
Triglyceride (mmol/L)	0.16	0.17	0.14	0.15	0.009	0.719	0.338	0.585	0.759
Cholesterol (mmol/L)	2.36	3.4	2.87	3.68	0.235	0.232	0.411	0.062	0.808
Alanine transaminase (U/L)	21.24	19.81	19.33	21.11	1.39	0.956	0.917	0.951	0.587
Total protein (g/L)	48.69	44.51	47.23	44.78	2.93	0.955	0.923	0.593	0.889
Albumin (g/L)	26.58	25.44	28.56	24.05	1.405	0.729	0.918	0.337	0.564
Urea (mmol/L)	3.78ab	5.06c	4.61ac	3.65b	0.183	0.010	0.357	0.621	0.001
HDL-C (mmol/L)	1.47	1.7	1.55	1.8	0.123	0.801	0.736	0.355	0.955
LDL-C (mmol/L)	1.37	1.99	1.68	2.08	0.117	0.117	0.370	0.028	0.636
Alkaline phosphatase (U/L)	165.8ab	122.1a	164.4ab	180.0b	8.35	0.073	0.080	0.376	0.067
Aspartate aminotransferase (U/L)	0.16	0.13	0.12	0.12	0.01	0.492	0.310	0.327	0.529

LN = Leymus as the only roughage with No calcium salts of fatty acid, LC = Leymus as the only roughage with Calcium salts of fatty acids, AN = Alfalfa replace 50% leymus with No calcium salts of fatty acids, AC = Alfalfa replace 50% leymus with Calcium salts of fatty acids.

LDL-C, low-density lipoprotein cholesterol; HDL-C, high-density lipoprotein cholesterol.

*p*-value: Treatment: *P* value of Duncan’s multiple comparisons between diets treatments aund different lowercase letters within the same row denote significant differences between the treatments; alfalfa, CASF and alfalfa×CSFA: the factorial analysis of crude protein levels, CSFA and their interaction.

#### Alfalfa and fat increase ammonia concentration and have additive effect in rumen fermentation

The results of rumen fermentation characteristics are shown in Table 3. Replacement *Leymus chinensis* with alfalfa significantly reduced the concentrations of NH_3_-N, acetate, isobutyrate, butyrate, total volatile fatty acid (TVFA) and Acetate/Propionate (*P* < 0.05), and increased the concentration of valerate (*P* < 0.05). The addition of CASF increased concentrations of ammoniacal nitrogen (NH_3_-N) isobutyrate, isovalerate and Acetate/Propionate in rumen (*P* < 0.05), and tended to raise valerate concentrations (*P* = 0.070). There were significant additive effects between alfalfa and CSFA on NH_3_-N, acetate, isobutyrate, butyrate, isovalerate and TVFA (*P* < 0.05). The concentration of NH_3_-N in LC group was significantly higher than that in other three groups (*P* < 0.05), and AN group was significantly higher than that in AC three group (*P* < 0.05). Acetate concentration in AN diet was significantly lower than that of other diets (*P* < 0.05). The concentration of valerate and Acetate/Propionate in LN group was significantly higher than that in other three groups (*P* < 0.05). The isobutyrate and butyrate concentrations in LC diet were significantly higher than those in other diet (*P* < 0.05). And the isobutyrate in AN diet were significantly higher than those in LN and AC diet (*P* < 0.05). The concentration of TVFA in LC group was significantly higher than that in AC group (*P* < 0.05).

**Table 3:** Effect of dietarys on rumen fermentation of Holstein bulls

Item	Dietary treatment					*p*-value			
	LN	LC	AN	AC	SEM	Treatment	alfalfa	CSFA	alfalfa × CSFA
NH_3_-N (mg/dL)	6.01ac	9.86b	6.8c	5.2a	0.344	<0.001	0.0007	0.0394	<0.001
pH	6.11	6.01	6.09	5.99	0.0329	0.5106	0.7812	0.139	0.9212
VFA (mmol/L)									
Acetate	67.15a	67.86a	64.87a	59.44b	0.739	<0.001	<0.001	0.088	0.027
Propionate	14.11	15.75	15.49	16.61	0.42	0.209	0.184	0.101	0.757
Isobutyrate	0.47a	0.56b	0.51c	0.48a	0.005	<0.001	0.027	0.002	<0.001
Butyrate	10.8ab	13.0c	11.7a	10.1b	0.19	<0.001	0.003	0.360	<0.001
Isovalerate	1.07a	1.26b	1.17c	1.15c	0.014	<0.001	0.991	0.001	<0.001
Valerate	0.56a	0.66b	0.68b	0.68b	0.014	0.004	0.010	0.070	0.056
TVFA	94.18ab	99.08a	94.38ab	88.46b	1.09	0.007	0.015	0.810	0.011
Acetate/Propionate	4.8a	4.35b	4.25b	4.05b	0.066	0.001	0.001	0.010	0.315

LN = Leymus as the only roughage with No calcium salts of fatty acid, LC = Leymus as the only roughage with Calcium salts of fatty acids, AN = Alfalfa replace 50% leymus with No calcium salts of fatty acids, AC = Alfalfa replace 50% leymus with Calcium salts of fatty acids.

*p*-value: Treatment: *P* value of Duncan’s multiple comparisons between diets treatments and different lowercase letters within the same row denote significant differences between the treatments; alfalfa, CASF and alfalfa × CSFA: the factorial analysis of crude protein levels, CSFA and their interaction.

#### CASF reduces cooking loss of the *Longissimus* muscle

*Longissimus* muscle, as one of the top grade beef, was used to describe the effects of various treatments on meat quality. *Leymus chinensis* and alfalfa had no impact on the meat quality (Table 4). CASF can significantly reduced cooking loss of the *Longissimus* muscle (*P* < 0.05). The cooking loss in AN group was significantly higher than that in LN and AC group (*P* < 0.05).

**Table 4:** Effect of dietarys on quality traits of Longissimus muscle

Item	Dietary treatment				SEM	*p*-value			
	LN	LC	AN	AC		Treatment	alfalfa	CSFA	alfalfa × CSFA
Shear force, kg	4.35	3.88	4.18	4.28	0.133	0.619	0.667	0.481	0.297
Cooking loss, %	33.7ab	32.5a	35.7b	31.2a	0.509	0.010	0.679	0.004	0.089
Water holding capacity, %	55.5	53.5	54.0	54.4	0.606	0.711	0.805	0.535	0.338
Drip loss, %	6.53	6.75	6.43	6.91	0.112	0.439	0.900	0.126	0.569
Dry matter, %	26.5	26.6	26.3	26.9	0.182	0.658	0.833	0.325	0.447
Crude protein, % DM	83.2	83.0	82.8	83.1	0.165	0.835	0.650	0.908	0.427
Intramuscular fat, % DM	14.6	14.9	15.0	14.8	0.171	0.858	0.683	0.920	0.447

LN = Leymus as the only roughage with No calcium salts of fatty acid, LC = Leymus as the only roughage with Calcium salts of fatty acids, AN = Alfalfa replace 50% leymus with No calcium salts of fatty acids, AC = Alfalfa replace 50% leymus with Calcium salts of fatty acids.

*p*-value: Treatment: *P* value of Duncan’s multiple comparisons between diets treatments and different lowercase letters within the same row denote significant differences between the treatments; alfalfa, CASF and alfalfa × CSFA: the factorial analysis of crude protein levels, CSFA and their interaction.

#### AC group has the highest Alpha diversity of ruminal bacteria among diet treatments

Total 32 samples were sequenced and reached 968470 high quality reads, average 30264 of each sample. When samples were normalized to 12556 reads depth, there generated 2237 operational taxonomic units (OTUs) by our analysis based on 97% similarity identity between reads. In order to estimate whether detected depth has reached the depth on behalf of rumen bacteria, the Good’s coverage was calculated, which result revealed that the sequenced reads was able to describe at least 96.9% of the rumen bacterial community. Mean Alpha diversity index (Table 5) indicated that the shannon index, Observed species and PD whole tree of AC dietary treatment were significantly higher than those of LC treatment (*P* < 0.05); meanwhile, observed species of LN group was higher when compared with LC treatment (*P* < 0.05), and with no difference with AC dietary treatment. There were no significant differences for four groups of Chao1.

**Table 5:** Mean richness estimates for dietarys

Item	Dietary treatment				SEM	*P*-value
	LN	LC	AN	AC		
Shannon index	8.10ab	7.79a	7.98ab	8.27b	0.06	0.029
Observed species	1137.64a	1029.2b	1087.03ab	1155.46a	17.96	0.0498
Chao1	1482.25	1380.9	1434.42	1491.17	19.22	0.154
PD whole tree	94.04ab	88.02a	91.81ab	95.99b	1.23	0.043

Even sampling depth based on sample with the lowest number of sequences (12556).

LN = Leymus as the only roughage with No calcium salts of fatty acid, LC = Leymus as the only roughage with Calcium salts of fatty acids, AN = Alfalfa replace 50% leymus with No calcium salts of fatty acids, AC = Alfalfa replace 50% leymus with Calcium salts of fatty acids.

*p*-value: *P* value of Duncan’s multiple comparisons between diets treatments and different lowercase letters within the same row denote significant differences between the treatments.

#### Taxonomic composition of bacterial communities in rumen

**Figure 1:**
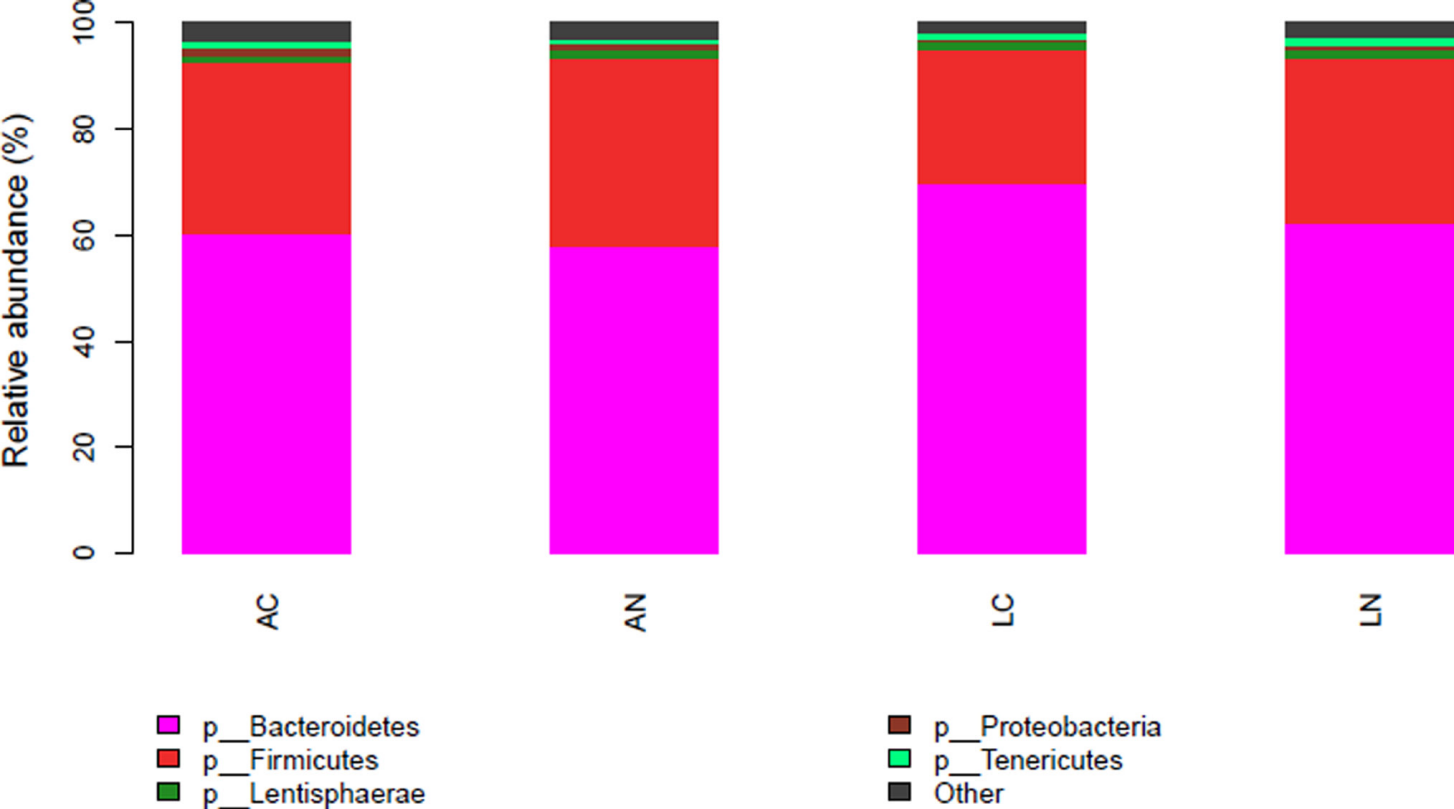
Effect of dietary treatments on phylum relative abundance of the ruminal bacterial community *N* = 8 for each treatment. LN = Leymus as the only roughage with No calcium salts of fatty acid, LC = Leymus as the only roughage with Calcium salts of fatty acids, AN = Alfalfa replace 50% leymus with No calcium salts of fatty acids, AC = Alfalfa replace 50% leymus with Calcium salts of fatty acids.

**Figure 2:**
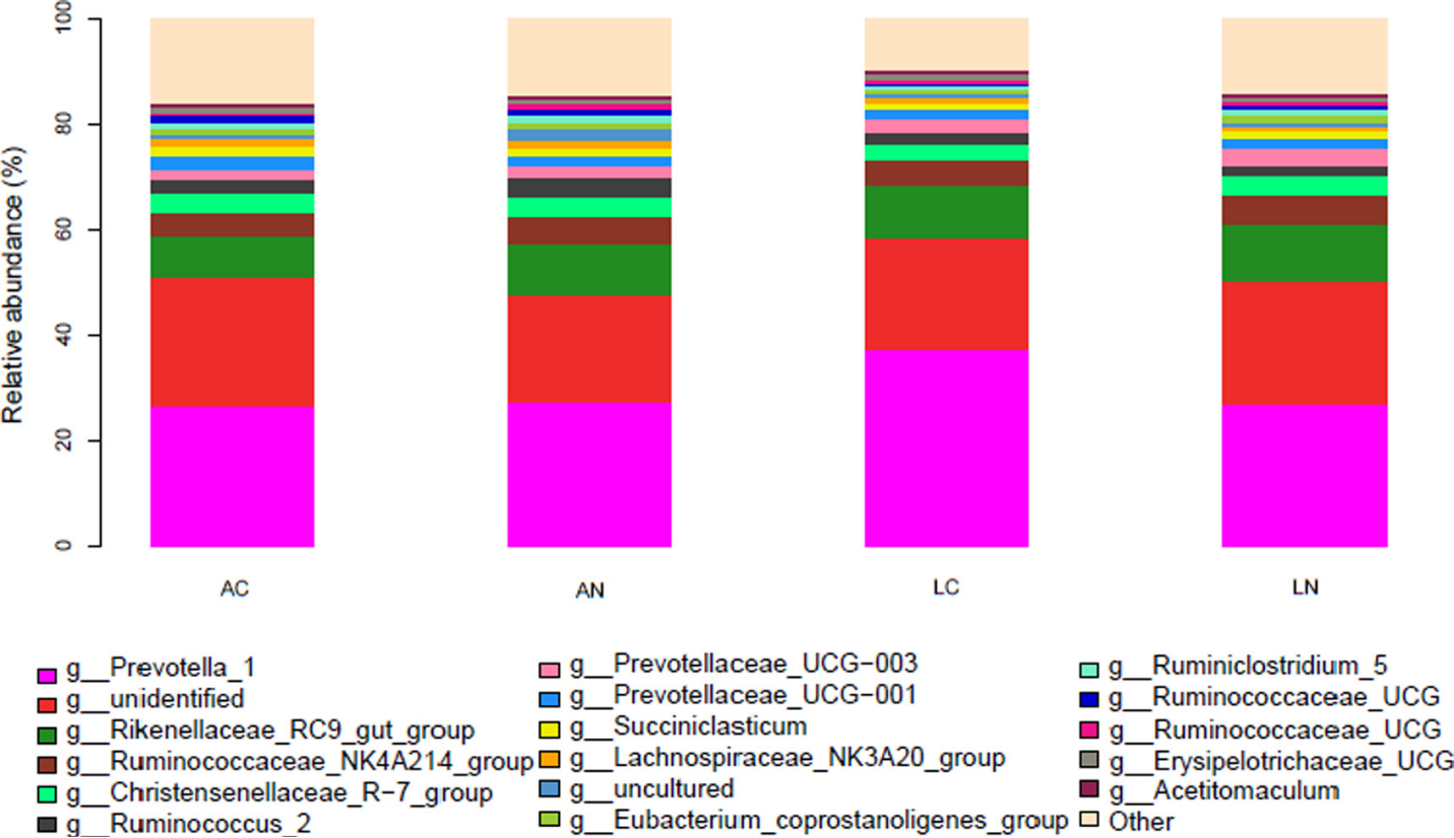
The effects of dietary treatments on the Genus (the relative abundance) of the ruminal bacterial community *N* = 8 for each treatment. LN = Leymus as the only roughage with No calcium salts of fatty acid, LC = Leymus as the only roughage with Calcium salts of fatty acids, AN = Alfalfa replace 50% leymus with No calcium salts of fatty acids, AC = Alfalfa replace 50% leymus with Calcium salts of fatty acids.

Based on 16S bacterial ribosomal databases of Silva, 20 phyla were distinguished in the rumen bacteria of all samples (Figure 1 and Supplementary Table 1). In the mean of all samples, the abundance of 5 phyla was >1%, and the dominant phyla were *Bacteroidetes* and *Firmicutes* , which abundances were 62.56% and 31.07%, respectively; the other 3 phyla abundance >1% were *Lentisphaerae* (1.36%), *Proteobacteria* (1.17%) and *Tenericutes* (1.14%). However, the other identified 15 phyla with relatively low abundance accounted for 2.72% of the total bacteria among all the samples, and the abundance of unidentified phyla accounted for 0.02% of the rumen bacterial flora. Kruskal-Wallis rank sum test was used to analysis of the significant differences between the diets group, the result indicated that the abundance of *Bacteroidetes* in LC diet was significantly higher than the other groups, but *Spirochaetae* in LC group was significantly lower than the other 3 groups (*P* < 0.05); the abundance of *SHA-109* phylum in AN group was higher than the other 3 groups. At the genus level, 77.66% of the OTUs were identified and the taxonomic analysis indicated that there were 2 main genera in the rumen bacteria (Figure 2 and Supplementary Table 2). *Prevotella_1* and *Rikenellaceae_RC9_gut_group* were the predominant genera accounting for 28.97% and 9.54% of the total samples, respectively. Moreover, there were 10 other relative abundance of genera is greater than 1%. According to the Kruskal-Wallis rank sum test, 12 identified genera were significant differences in the 4 groups, including genus *Treponema_2* which average relative abundance is greater than 0.5%.

#### No obviously clustered was observed among the groups by PCoA

**Figure 3:**
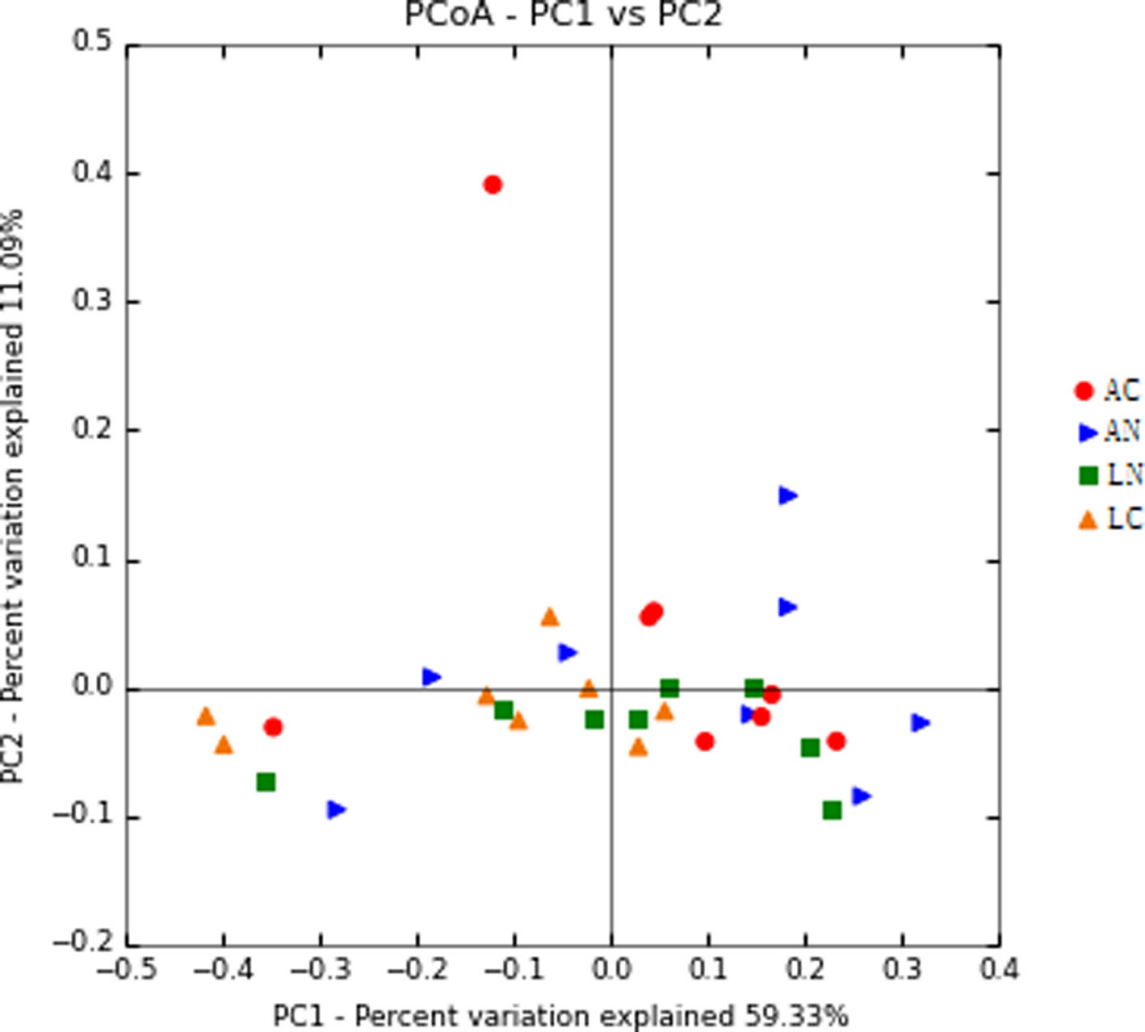
Principal Coordinate Analysis (PCoA) of rumen bacterial community structures of the four dietary treatments The PCoA plots were constructed using the weighted UniFrac method. *N* = 8 for each treatment. LN = Leymus as the only roughage with No calcium salts of fatty acid, LC = Leymus as the only roughage with Calcium salts of fatty acids, AN = Alfalfa replace 50% leymus with No calcium salts of fatty acids, AC = Alfalfa replace 50% leymus with Calcium salts of fatty acids.

In this study, beta diversity was estimated by principal co-ordinates analysis (PCoA ) based on the weighted unifrac distances and shown that the samples with no obviously clustered according to dietary groups (Figure 3). PC1 and PC2 explained 59.33% and 11.09% of variation, respectively.

Correlation between the relative abundances of ruminal bacterial genera and rumen fermentation characteristics, nutrients apparent digestibility, serum biochemical parameters

**Figure 4:**
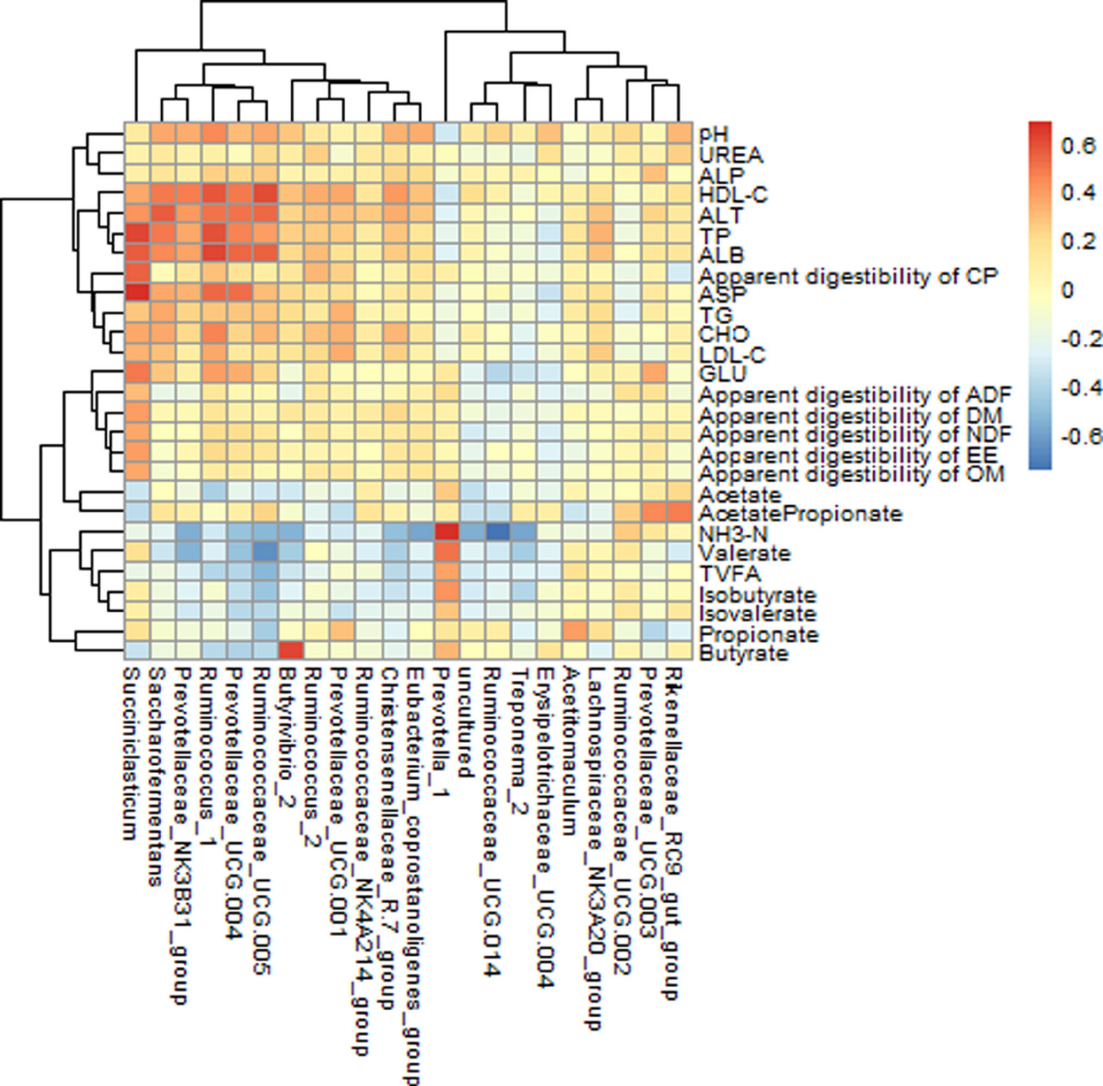
Correlation between bacterial genus abundance and rumen fermentation characteristics, nutrients apparent digestibility, serum biochemical parameters *N* = 32 for each point. Only the predominant bacterial genera (relative abundance ≥0.1% in each sample) are presented; Cells are colored based on spearman rank correlation test between rumen bacteria and rumen fermentation characteristics, nutrients apparent digestibility, serum biochemical parameters. The red color represents a positive correlation, the blue color represents a negative correlation.

The rumen bacterial genera, which relative abundances were more than 0.1% in all 32 sequenced samples, were used to calculate correlation with other indicators, and the results were shown in Figure 4. In this present study, if the correlation coefficient *r* > 0.35, we considered they were correlated with each other between the two indicators (*p* < 0.05). Valerate concentration in rumen had a strong negative correlation with the abundance of *Ruminococcaceae_UCG-005* (*r* = −0.666; *P* < 0.001). Butyrate had a strong positive correlation with the abundance of *Butyrivibrio_2* (*r* = 0.619; *P* < 0.001); NH_3_-N had a strong positive correlation with the abundance of *Prevotella_1* (*r* = 0.667; *P* < 0.001); while, it also had a strong negative correlation with the abundance of *uncultured* (*r* = –0.569; *P* < 0.001), *Butyrivibrio_2* (*r* = −0.556; *P <* 0.001), *Treponema_2* (*r* = –0.598; *P <* 0.001), *Eubacterium_coprostanoligenes_group* (*r* = –0.595; *P <* 0.001), *Prevotellaceae_NK3B31_group* (*r* = −0.570; *P <* 0.001), *Ruminococcaceae_UCG-014* (*r* = −0.761; *P* < 0.001). ALT concentration in serum had a strong positive correlation with the abundance of *Saccharofermentans* (*r* = 0.556; *P <* 0.001). Total protein (TP) concentration in serum had a strong positive correlation with the abundance of *Ruminococcus_1* (*r* = 0.588; *P <* 0.001). Albumin (ALB) concentration in serum had a strong positive correlation with the abundance of *Succiniclasticum* (*r* = 0.556; *P* < 0.001). HDL-C concentration in serum had a strong positive correlation with the abundance of *Ruminococcus_1* (*r* = 0.567; *P <* 0.001) and *Ruminococcaceae_UCG-005* (*r* = 0.597; *P <* 0.001). Aspartate aminotransferase (ASP) concentration in serum had a strong positive correlation with the abundance of *Succiniclasticum* (*r* = 0.677; *P* < 0.001).

## DISCUSSION

Rumen has an important role on physiological function in ruminants for the degradation of nutrients, especially plant fibers. Microbial fermentation in rumen was influenced by environmental, genetics and body condition of the host [14], which also directly or indirectly impact on the host to degrade, digest and absorb nutrients [15]. Therefore, the diet can alter the composition of rumen microorganisms, which potentially promote the growth of beef cattle. Fat deposition is the main purpose for beef cattle in the latter finishing period, therefore, feed with high energy density is often used during this period. In general, CASF and the alfalfa are usually used for dairy production, and the CASF as rumen-protected fat might be more conducive to fat deposition. However, limited studies have been carried out in beef production. So, the aim of this study is to assess the effects of CASF and alfalfa in beef cattle production, including growth, feed intake, apparent digestibility, serum biochemical parameters, rumen fermentation characteristics as well as bacterial communities.

Alfalfa supplementation improved feed conversion ratio with no effect on DM intake in the present study, and reduced the formation of acetate and butyrate fermentation in the rumen. That means more nutrients directly passed the rumen without fermentation, leading the higher nutrient digestion and absorption in digestive tract. However, alfalfa used in this experiment had high ADF, which caused that the digestive enzymes cannot combine with substrates, consequently, resulted in alfalfa had no significant effect on apparent digestibility of nutrients. The alfalfa diet did not improve the meat quality of *Longissimus*, which were in similar results with other studies [8, 16]. Fiber-degrading bacteria can use ammonia as the nitrogen sources for growth [17]. Increasing the concentration of ammonia in the rumen is conducive to bacterial growth, which is beneficial for cellulose fibers to produce acetate and butyrate [18]. This is why alfalfa reduced the concentration of NH_3_-N and also acetate, butyrate, TVFA and Acetate/Propionate in rumen.

Fatty acids are absorbed in the duodenum [19] and improved the growth performance, meat quality and health of beef cattle. Generally, dietary fat can reduce dry matter intake, nutrients apparent digestibility, particular digestibility of crude fiber, because high fat diets has adversely influence on rumen microorganism [1, 20]. The CSFA as a rumen-protected fat had no effect on the apparent digestibility of nutrients in the present study. However, the CASF can inhibit the rumen fermentation and reduce the ratio of Acetate/Propionate, which is consistent with studies on the dairy [19]. Simultaneously, we also found that the CASF can increase the branched-chain volatile fatty acid (isobutyrate and isovalerate), which are generated by rumen fermentation and decomposition of branched-chain amino acids. The reason for this result was that the CASF increased NH_3_-N concentration in rumen and changed the characteristics of rumen microbial fermentation [21]. In serum biochemistry, CASF can increase the concentration of cholesterol and triglyceride in the liver through the synthesis of fat metabolism [3], which can elevate serum cholesterol levels. We observed that it significantly increased LDL-C content, which indicated that the CASF supplementation is detrimental to the health of cattle, because of increasing the risk of atherosclerosis.

Compared with LN group, the increases in the apparent digestibility of DM, CP, NDF and OM in LC group were the results of that CASF is a salt, which are not completely inert in the rumen, and can release a part of fatty acids in the rumen, then interacting with the rumen environment and being hydrogenated by rumen microorganism [22]. This mechanism is not caused by the increase of calcium in diets, possibly because that the CSFA increased the proportion of C16:0 in the diet which was not harmful to rumen microbes, while the unsaturated fatty acids in CASF can improve cholecystokinin release in the intestine [23]. They led to increased digestibility of nutrients, but no effect on ADF apparent digestibility. Dietary supplementation of fat is beneficial to the energy and nitrogen balance of ruminant, which contributes to the fermentation of rumen [24]. Moreover, the CSFA can effectively avoid the harmful effects of fatty acids on cellulolytic bacteria.

Alfalfa instead of *Leymus chinensis* increased fiber content in diets, which was not conducive to the combination of digestive enzymes and substrates, and hindered the combination of calcium and fatty acids released from fatty acid calcium salts. The results showed that the alfalfa and CSFA had antagonistic additive effects on apparent digestibility of nutrients. At the same time, this antagonistic effect also inhibited the rumen fermentation, because these released fatty acids can restrain the fermentation of microorganisms, fatty acids inhibited the growth of fiber-degrading bacteria and reduced the production of acetic acid.

Fatty acids are absorbed into the intestinal cells and blood, which is inseparable from the transport of lipoprotein, especially long-chain fatty acid transport needs more lipoprotein transport system for mobilization [25]. The synthesis of lipoproteins requires the absorption of amino acids, which has similar protein levels in the treatment group, and therefore, dietary supplementation of CSFA increased the amino acids requirements for the synthesis of lipoproteins. The fatty acid absorption has synergism to amino acids absorption.

In this study, Good’s coverage of all samples was evaluated and reached 0.967, which indicated that the study has covered 96.7% OTU calculated in a 97% similarity. Because diets are major factors on the rumen microbial fermentation [26], then this research just directly compared four diets on rumen microbial effects, without using factorial analysis to individually assess the effects of alfalfa, CASF and their additive effects, respectively. Alpha diversity indices revealed a higher diversity in AC diet compared to LC diet. However, the diversity between LN and AN diets had no difference, which suggested that different combinations of feed ingredients produced different biodiversity in rumen even though they have similar nutritional composition, and that AC and LC dietary treatments hosted a distinct bacterial community with regard to bacterial makeup. However, in our study, the nutrients in the diet of various groups are equal except to adding calcium salts of fatty acids, which led that there were no significantly cluster among 4 treatment groups of beta diversity estimated by PCoA and proved they have similar bacterial community.

Bacteria as the main composition of gastrointestinal microbes [27], plays an important role on the rumen function. It synthesized necessary amino acids and vitamins with non-protein nitrogen, and generated volatile fatty acids by decomposing cellulose and starch to provide energy to the host [28]. Therefore, the rumen bacterium as a complex micro-ecological composition, changes of diet can impact rumen microbial metabolism, ultimately changing host performance and product quality. The composition of rumen bacteria community showed that the bacterial community was predominated by *Bacteroidetes* and *Firmicutes*, which distributed similarly with Jami [29], and *Bacteroidetes* has the highest relative abundance than the others. But behind the two dominant phyla the others variation is very large. Generally, *Proteobacteria* is the third-largest phylum in the rumen [29, 30], but it was ranked behind *Lentisphaerae* in this study. However, Zhang *et al.* [31] found that the *Tenericutes* is the third-largest phylum. Such different results may be due to the relative abundance of these phyla are very small, and vulnerable to diets and the effects of other factors. At the phylum level, *Bacteroidetes* relative abundance of LC group was significantly higher than AN group, because their diet composition are differences in maximum, and fatty acids can inhibit the growth of numerous bacteria [32]; meanwhile, it also means that exists additive effects between alfalfa and CASF. It is generally believed that a greater diversity of rumen microbes flora are benefit for the stable of rumen environment [33], suggesting that LC dietary was more advantageous to stability of rumen micro-ecological environment.

At genus level, in the relative abundance of more than 1% bacteria, there were no significant differences among the four diets groups, meanwhile, PCoA also verified that one of the results. All available reports suggested that *Prevotella* genus is the dominant ruminal genus in abundance [26, 34], indicating that *Prevotella* is stable in the rumen and not easily influenced by experimental factors. Stevenson *et al.* reported that it has the rate-limiting performance of dipeptidase activity [35], suggesting it takes charge of hydrolysis oligopeptides. Therefore, *Prevotella* has an important role in degradation of protein in rumen, especial in breakdown dipeptide. In this study, *Rikenellaceae_RC9* was included in the most abundant ruminal bacteria genera. *Ruminococcus* and *Fibrobacter* genera belong to *Firmicutes* phylum have the function of cellulosis [26]. The relative abundance of *Ruminococcus* genus accounts for 2.52% of all the rumen bacteria regardless of dietary treatments, which fits with the results of Bainbridge *et al.*, who showed that *Ruminococcus* accounts for 2.59% of bacteria in rumen of Holstein cows. However, there were different reports that the level was higher [36] or lower [26] than this level. *Ruminococcaceae* family contains several genera, in which *Ruminococcaceae_NK4A214_group* with the highest relative abundance and about 5.1% of total bacteria. Whereas, *Prevotellaceae_UCG-003* genus and *Prevotellaceae_UCG-001* genus are part of *Prevotellaceae* family which belong to *Bacteroidetes* phylum and have the function of breakdown hemicellulose and protein [37]. *Succiniclasticum* genus, which belongs to *Firmicutes* phylum can generate propionate by degrading the succinate [38]; and it represented about 1.52% of total bacteria in this study. As a whole, dominant microflora as main performer in rumen fermentation and were not significantly different among the four treatment groups, eventually led to the apparent digestibility of nutrients in the diet had no significant difference.

It has been reported that host serum biochemical index, immunization and health were influenced by the gastrointestinal tract microorganisms [39, 40]; meanwhile, microbial structure, function and abundance work on the host’s phenotype. In terms of rumen fermentation characteristics, there were extremely significant correlations between rumen NH_3_-N concentration and variety of Bacterial genera, because NH_3_-N is wholly generated and utilized by microbial metabolism in rumen. Such as NH_3_-N was significantly positively correlated with *Prevotella_1* genus, because of its degradation protein activity was advantageous to produce NH_3_-N; however, *Butyrivibrio_2*, *Prevotellaceae_NK3B31_group* and *Ruminococcaceae_UCG-014* genera as degradation of cellulose and hemicellulose bacteria, which can synthesis bacterial protein by using non-protein nitrogen, and resulting in them was negatively correlated with the NH_3_-N concentration. Similarly, *Butyrivibrio_2* genus can produce butyrate by the degradation of plant cellulose and starch in the rumen. Because it can generate mucosal butyrate and release butyrate close to the epithelial tissue, and enhance biological availability of butyrate for the ruminant [41], thus, there was a significant positive correlation between *Butyrivibrio_2* genus and butyrate. Serum biochemical parameters associated with many bacterial genera in the rumen in this study. There was no evidence for rumen bacteria directly act on the serum biochemical parameters. The possible causes of this correlation is that the degradation products of rumen bacteria through the hepatic metabolism to change serum biochemical parameters.

In summary, alfalfa supplementation can improve feed conversion ratio during the finishing period of beef cattle. Dietary CASF increased the content of serum LDL-C, and reduced the cooking loss of beef. CASF and the alfalfa altered rumen fermentation, while had no significant effects on apparent digestibility of nutrients. Alfalfa and CASF had significant additive effects on the apparent digestibility of nutrients and rumen fermentation. In rumen bacteria, compared with additional alfalfa, dietary adding CASF increased microbial diversity index, but no significant difference in bacteria abundance at the genus level was found among the four dietary treatments. In correlation analysis, the relative abundances of rumen bacterial genera not only had correlation with apparent digestibility of nutrients and rumen fermentation characteristics, but also had correlation with serum biochemical parameters. Overall, the results of this study suggested that the application of CASF and alfalfa would be beneficial in beef cattle production.

## MATERIALS AND METHODS

### Ethical approval

The cattle experiment was approved by the Ethics Committee of China Agricultural University (Permit No. DK1008), and in accordance with the guidelines and regulations that enacted by the Administration of Affairs Concerning Experimental Animals.

#### Experimental design and animal diet

A total of 48 Holstein bulls at 20 months of age were selected and randomly divided into four treatments. Each treatment contained 12 bulls. From birth to beginning of this trial, these bulls received the same diet. One of four diets provided to each group: 1) basic diet: *Leymus chinensis* as the only roughage with no CASF (LN); 2) alfalfa replace 50% *Leymus chinensis* with no CASF(AN); 3) basic diet with 2.4% CASF(LC); 4) Alfalfa replace 50% *Leymus chinensis* with 2.4% calcium salts of fatty acid (AC). Feed formula and nutrition components of the trial are shown in Table 6. All cattle were fed twice a day at 7:30 and 17:30, respectively. All animals were fed *ad libitum* and allowed about 10% orts each day, feed intake was recorded every day. Fresh drinking water was offered free-choice at all times. During the 90 days trial period, body weight of cattle was recorded monthly. Feed intakes and orts were recorded daily.

**Table 6:** Ingredients and nutrient compositions of the dietary treatments^1^

Item	LN	LC	AN	AC
Ingredient, % (DM basis)				
corn grain	40.44	39.47	38.16	37.24
soybean meal	8.22	8.02	6.72	6.56
wheat grain	9.84	9.6	13.62	13.29
Leymus chinensis	40	39.04	20	19.52
Alfalfa	0	0	20	19.52
Calcium salt of fatty acids	0	2.4	0	2.4
NaCl	0.6	0.59	0.6	0.59
Mineral-vitamin premix^2^	0.6	0.59	0.6	0.59
Limestone	0.3	0.29	0.3	0.29
Chemical composition, % (DM basis)				
OM	94.07	93.18	94.26	93.72
CP	12.84	12.54	12.42	12.16
NDF	42.63	42.96	42.45	42.91
ADF	18.4	18.26	21.01	20.94
EE	6.71	6.69	6.48	6.5
Main fatty acids profile, % of total				
C16:0	16.8	28.4	17.1	28.2
C18:0	2.7	3.2	2.6	3.3
C18:1n-9 *cis*	26.3	27.8	26.3	27.8
C18:2n-6 *cis*	49.3	36.0	49.1	36.0
C20:1	3.3	2.7	3.1	2.6

^1^Dietary treatments: LN = Leymus as the only roughage with No calcium salts of fatty acid, LC = Leymus as the only roughage with Calcium salts of fatty acids, AN = Alfalfa replace 50% leymus with No calcium salts of fatty acids, AC = Alfalfa replace 50% leymus with Calcium salts of fatty acids.

^2^Every kilogram of mineral–vitamin premix contained: 1000000 IU Vitamin A, 350000 IU Vitamin D3, 5.7 g Zn, 4.2 g Fe, 5.72 g Mn, 2.5 g Cu, 85 mg I, 85 mg Se, and 30 mg Co.

OM, organic matter; CP, crude protein; NDF, neutral detergent fiber; ADF, acid detergent fiber; EE, ether extracts; DM: dry matter.

#### Samples collection and treatment

Feed and orts samples were collected monthly. Feces were gathered at 6:00, 12:00, 18:00 and 24:00 on continuous 3 days, and 10% tartaric acid solution was added into the feces, 65. Approximate 10 mL of blood was collected through jugular vein at 7:00 in the morning, then centrifuged at 3000 g for 15 min to collect supernatant serum and stored at −20°C. Two hours after the morning feeding, 100 mL ruminal fluid was collected by using esophageal tubing and discarded the first 300 mL to avoid cross contamination of saliva and the samples. All cattle were slaughtered at the end of the feeding experiment. After chilled 4°C for 48 hours, about 500 g and 5 cm length of *Longissimus* muscle were taken at the left half-carcass between the 6 and 7 ribs.

#### Chemical analysis of samples

The ether extract (EE), DM, crude ash and CP of feed, orts and feces were analyzed by using the methods of AOAC [42]. Acid-insoluble ash was measured according to previous methods [43], and used as an endogenous indicator to calculate the apparent digestibility of nutrients. Glucose (GLU), cholesterol, alanine transaminase (ALT), total protein, albumin, urea, high density lipoprotein cholesterol, low density lipoprotein cholesterol, alkaline phosphatase and aspartate aminotransferase (ASP) of serum were measured by Automatic biochemical Analyzer (HITACHI 7020, Tokyo, Japan) and the corresponding kits (Strong Biotechnologies Inc, Beijing, China). Volatile fatty acids (VFA) of rumen fluid were tested by gas chromatography (Shimadzu GC-2014, Kyoto, Japan), and referenced the prior conditions [44]. The NH_3_-N concentration in the rumen liquid was determined according to previous methods [45] using a spectrophotometer (UV-1700, Shimadzu Corporation, Kyoto, Japan)

A total of 32 rumen content samples, randomly selected 8 Holstein bulls from each group, were collected at 10:00 via esophageal tubing [30]. The 16sDNA of rumen content samples were extracted using the bacterial DNA Kit (Omega Bio-Tek, Norcross, GA, USA) according to the manufacturer’s protocol. DNA concentration and purity were determined on a UV-1700 spectrophotometer (Shimadzu, Kyoto, Japan). The V3-V4 region of the bacteria 16S rRNA gene were amplified with the universal primers of the forward 338F(5′-ACTCCTACGGGAGGCAGCAG-3′) and the reverse 806R (5′-GGACTACHVGGGTWTCTAAT-3′), and subjected to high-throughput sequencing by Beijing Allwegene Tech, Ltd (Beijing, China) using the Illumina Miseq PE300 sequencing platform (Illumina, Inc., CA, USA). These primers contained a set of 8-nucleotide barcodes sequence unique to each sample. The PCR program was as follows 95°C for 5 min, 25 cycles at 95°C for 30 s, 55°C for 30 s, and 72°C for 30 s with a final extension of 72°C for 10 min. DNA amplification products were retrieved by 2% agarose gels and purified using the AxyPrep DNA Gel Extraction Kit (Axygen Biosciences, Union City, CA, U.S.) according to the manufacturer’s instructions and quantified using QuantiFluor™ -ST (Promega Corporation, Madison, WI, U.S.). Purified amplicons were pooled in equimolar and paired-end sequenced (2 × 300) on an Illumina MiSeq platform (San Diego, CA, USA) according to the standard protocols.

#### Processing of sequencing data

Raw quality control of the generated sequences was executed by using the Trimmomatic and Usearch Software, which included trimming of the 3′ end of sequences that dropped below the average 20 score over a 50 bp and removing sequences with unidentified bases, while chimeric sequences were identified and removed using Usearch Version 8.1.1861 in the method of UPARSE [46]. Resulting sequences were demultiplexed using the QIIME software package (version 1.9.1) [47]. Subsequently, sequences were binned into OTUs at 97% similarity using the UPARSE pipeline (USEARCH Version 8.1.1861) [46]. Representative sequences from each OTU were assigned taxonomy by using the RDP Classifier [48] method against the Silva119 [49] 16S rRNA database as reference sequences using confidence threshold of 70%.

#### Statistical analysis

All of the data were analyzed by proc GLM of SAS version 9.0 software (SAS Institute Inc., Cary, NC, USA) in a 2 × 2 factorial arrangement with a complete randomized design by the model: Y_ij_ = μ+A_i_+C_j_+(AC)_ij_ +e_ij_, where Y_ij_ is the dependent variable, μ is general mean, A_i_ is fixed effect of alfalfa (i = 0 or 1), C_j_ is the fixed effect of CSFA (j = 0 or 1), (AC)_ij_ is the additive effects between alfalfa and CSFA, and e_ijk_ is the residual effect. The normality and homogeneity of the variance were checked using proc UNIVARIATE. The differences in the dietary treatments were evaluated by Duncan’s multiple range tests when *p* < 0.05 as the criterion of significance.

OTU of all samples were rarefied to the minimum sample depth (12556 reads) based on the Mersenne Twister pseudorandom number generator of QIIME. Alpha diversity estimators Chao1 and observed OTUs and rarefaction curves for the overall bacterial community and the weighted UniFrac distance matrix were calculated by using QIIME. PCoA based on weighted UniFrac distances was operated to compare all samples, and a non-parametric factorial Kruskal-Wallis sum-rank test was used to evaluate bacterial phylum and genus differences among samples.

Correlation analysis was counted of between rumen bacteria and rumen fermentation characteristics, nutrients apparent digestibility, serum biochemical parameters by spearman rank correlation test using R software. Significance was declared at *P* < 0.05.

## SUPPLEMENTARY MATERIALS TABLES





## Abbreviations

CSFA: calcium salt of long-chain fatty acids
TVFA: total volatile fatty acids
DM: dry matter
OM: organic matter
CP: crude protein
EE: ether extract
NDF: neutral detergent fiber
ADF: acid detergent fiber
NH_3_-N: ammonia nitrogen
VFA: volatile fatty acid
LDL-C: low density lipoprotein cholesterol
HDL-C: high density lipoprotein cholesterol
PCoA: principal co-ordinates analysis
