# Supplementary material for: Effect of calcium salt of long-chain fatty acids and alfalfa supplementation on performance of Holstein bulls
Source: Oncotarget. 2017 Dec 9;9(3):3029–42. doi: 10.18632/oncotarget.23073 (PMC5790443; doi:10.18632/oncotarget.23073)
Supplement: Supplementary file 1 [file oncotarget-09-3029-s001.pdf]

Effect of calcium salt of long-chain fatty acids and alfalfa supplementation on performance of Holstein bulls

SUPPLEMENTARY MATERIALS

Supplementary Table 1: Effect of dietary treatments on the phylum of the ruminal bacterial community

ID	Dietary treatment				*p*_value
	AC	AN	LC	LN	
p__Bacteroidetes	0.600235	0.566433	0.688562	0.619888	0.041
p__Firmicutes	0.321330	0.363879	0.259503	0.314183	0.122
p__Lentisphaerae	0.010468	0.012050	0.015753	0.017232	0.134
p__Proteobacteria	0.019479	0.016708	0.008115	0.008533	0.816
p__Tenericutes	0.013843	0.012542	0.008614	0.012964	0.763
p__Spirochaetae	0.009860	0.007865	0.004840	0.009670	0.013
p__Fibrobacteres	0.007196	0.004463	0.003590	0.006164	0.510
p__Actinobacteria	0.008059	0.005770	0.003260	0.002758	0.139
p__Cyanobacteria	0.003083	0.003007	0.003373	0.002122	0.541
p__Saccharibacteria	0.001856	0.002536	0.001609	0.002016	0.609
p__Candidate_division_SR1	0.001671	0.001794	0.000653	0.001781	0.094
p__Elusimicrobia	0.001153	0.000869	0.001254	0.001254	0.724
p__SHA-109	0.000906	0.001372	0.000159	0.000717	0.014
p__Synergistetes	0.000211	0.000271	0.000134	0.000189	0.449
p__unidentified	0.000307	0.000168	0.000103	0.000162	0.456
p__Chloroflexi	0.000145	0.000137	0.000222	0.000193	0.790
p__Planctomycetes	0.000124	0.000018	0.000108	0.000071	0.252
p__Euryarchaeota	0.000035	0.000062	0.000080	0.000023	0.193
p__Verrucomicrobia	0.000028	0.000044	0.000067	0.000058	0.566
p__Fusobacteria	0.000012	0.0000036	0	0.0000164	0.432
p__Armatimonadetes	0	0.0000094	0	0.0000037	0.251

Supplementary Table 2: Effect of dietary treatments on the genus of the ruminal bacterial community. See Supplementary_Table_2
